# Associations of multimorbidity with body pain, sleep duration, and depression among middle-aged and older adults in China

**DOI:** 10.1186/s12955-024-02238-x

**Published:** 2024-02-27

**Authors:** Xin Ye, Xinfeng Wang

**Affiliations:** 1 Institute for Global Public Policy, Fudan University, 220 Handan Road, Yangpu District, 200433 Shanghai, China; 2 LSE-Fudan Research Centre for Global Public Policy, Fudan University, 220 Handan Road, Yangpu District, 200433 Shanghai, China

**Keywords:** Multimorbidity, Body pain, Sleep duration, Depression, Middle-aged and older adults

## Abstract

**Background:**

Multimorbidity, body pain, sleep disturbance, and depression are major clinical and public health challenges. This paper aimed to examine the associations of multimorbidity with body pain, sleep duration, and depression; and whether the associations varied by socioeconomic status.

**Methods:**

Data was derived from four waves of the nationally representative China Health and Retirement Longitudinal Study (CHARLS), including participants aged 45 years and older in 2011. 12 physical non-communicable diseases and 1 mental chronic disease were used to measure multimorbidity. Educational attainment and annual per-capita household consumption expenditure were employed as proxies for socioeconomic status.

**Results:**

Of the 16,931 participants aged 45 + years old, the proportion of people with multimorbidity was 37.87% at baseline. The number of multimorbidity increased with older age and higher socioeconomic status. Multimorbidity was associated with more body pain (incidence rate ratio (IRR) = 1.53, 95% CI = 1.45–1.61), and decreased sleep duration (β = -0.26, 95% CI = -0.36–-0.15). Furthermore, multimorbidity was associated with increased depression risks (odds ratio (OR) = 1.54, 95% CI = 1.44–1.64, adjusted for sociodemographic variables), with the mediating effects of the number of body pain and sleep duration. The associations between multimorbidity and depression persisted among different socioeconomic groups.

**Conclusions:**

Multimorbidity was associated with increased body pain, decreased sleep duration, and further led to increased depression risks. It is necessary to pay attention to the multimorbidity of middle-aged and older adults, relieve their body pain, guarantee sufficient sleep, so as to reduce depression risks.

**Supplementary Information:**

The online version contains supplementary material available at 10.1186/s12955-024-02238-x.

## Introduction

Multimorbidity is defined as the coexistence of two or more chronic conditions in an individual [1]. Chronic conditions are a major cause of health burden and inequalities in health outcomes in China [2, 3]. Literature shows that multimorbidity is highly prevalent among middle-aged and older population, ranging from 30 to 95% across age groups and countries [4]. In China, multimorbidity has a prevalence rate of nearly 50% among middle-aged and older adults [5, 6]. With an ageing population and elevated risk factors for non-communicable diseases, the prevalence of multimorbidity is likely to increase rapidly [7]. Meanwhile, depression, as a common clinical mental disorder, is one of the main causes of emotional distress in late life [8]. Previous studies have linked depression to a variety of chronic conditions [9]. However, its underlying mechanisms are not fully understood and need to be further explored.

Body pain is one of the most common symptoms in middle-aged and older adults. Results from a previous research based on China Health and Retirement Longitudinal Study (CHARLS) suggested that the prevalence of pain among people aged 45 years or older was 28.62% in 2015. Older people tended to have higher prevalence of pain [10], and the prevalence of chronic pain among people over 60 years old could reach 55% [11]. Pain intensity is closely related to depression of middle-aged and elderly. People with chronic pain had 4.1 times the risk of depressive symptoms compared to those without pain [12]. Another study showed that depressed patients were more likely to have chronic pain as compared with those non-depressed [13].

Sleep problems have become one of the major health challenges for middle-aged and elderly, and its close associations with depression and multimorbidity have been proposed by many researchers. As people age, they experience degenerative changes in their central nervous system, making it harder to fall asleep and stay asleep [14]. Sleep deprivation was associated with higher odds of multimorbidity than good sleep [15]. For middle-aged and older adults, depressive emotion may be associated with accelerated age-related structural changes in sleep [16]. Therefore, it is necessary to explore the mediating role of body pain and sleep duration in the association between multimorbidity and depression.

Overall, limited evidence on the health implications of multimorbidity is mainly from high-income countries. They explored pairwise relationships among multimorbidity, body pain, sleep duration, and depression. It was suggested that multimorbidity was associated with chronic pain [13, 17], sleep duration [15], and further correlated with depression [17, 18]. However, the comprehensive associations have not been widely examined in low- and middle-income countries (LMICs). Relevant research is crucial for understanding multimorbidity and informing targeted interventions. It can also guide policymakers and healthcare providers in effectively allocating limited resources to improve health outcomes among vulnerable populations.

To date, published studies were at the regional or district level and mostly used cross-sectional designs. This is the first study from China employing national panel survey data to examine the relationship between multimorbidity and depression, with the mediating role of body pain and sleep duration. Additionally, given that the possible health differences may vary by socioeconomic status [19], we also aim to explore whether the associations varied by socioeconomic status. Our study is expected to facilitate the identification of multimorbidity and the prevention of depressive symptoms in middle-aged and older adults.

## Methods

### Data and sample

For this population-based panel data analysis, data were derived from four waves of the CHARLS 2011, 2013, 2015 and 2018. All information included in our study was self-report via face-to-face interviews with a structured questionnaire, from a nationally representative sample of Chinese residents aged 45 years and older, using multistage stratified probability-proportionate-to-size sampling. The overall response rate for the first wave of CHARLS was 80.5%. The CHARLS data included overlapping individuals across the waves. The total sample size for the 2011 CHARLS baseline survey was 17,708 individuals, who were followed up every 2–3 years to repeat the survey. New respondents were also added to each follow-up survey. A detailed description of the objectives and methods of CHARLS has been reported elsewhere [20]. For this study, 16,931 participants aged 45 years and older at 2011 baseline were included, of which 2,647, 3,029, and 4,587 participants were lost to follow-up at each wave, respectively.

### Variables

#### Multimorbidity

In this study, multimorbidity was defined as the presence of two or more chronic non-communicable diseases [7]. 13 self-reported non-communicable diseases were used to measure multimorbidity, including diabetes, hypertension, dyslipidemia, heart disease, stroke, cancer, chronic lung disease, digestive disease, liver disease, kidney disease, arthritis, asthma, and psychological problems. The number of non-communicable diseases for each participant was added up to determine who had multimorbidity in each wave [21]. It is a categorical variable with the options “yes” (having two or more diseases) or “no” (having no or only one disease).

It is worth noting that, psychological problems originally refer to a broader range of psychological issues beyond depression, including emotional, nervous, and psychiatric problems. However, depression has been astonishingly ignored by Chinese older people and they seldom regard depression as a kind of disease [22], so psychological problems mainly denote to other psychiatric issues except depression. Furthermore, it is important to recognize that depression can be encompassed within psychotropic problems, and that stroke may induce vascular depression. In our study, the prevalence of psychotropic problems is relatively low (As shown in Supplementary Table 1), which supports our claim that depression is underestimated in China.

#### Body pain

In each wave of CHARLS, participants were asked, “Do you often suffer from body pain?” Those who answered “yes” were then asked, “On what part of your body do you feel pain? Please list all parts of the body where you are currently experiencing pain.” Participants were given several options for body parts (head, shoulders, arms, wrists, fingers, chest, stomach, back, waist, buttocks, legs, knees, ankles, toes, and neck). The number of pain sites greater than 1 was defined as multiple pain points.

#### Sleep duration

Self-reported sleep duration was obtained via a structured questionnaire that asked, “In the past month, how many hours have you actually slept at night (average hours for one night)?” and “In the past month, how long have you taken a nap after lunch?”. Hours of sleep at night and after lunch were added to derive sleep duration in each wave.

#### Depression

The dependent variable was depression. The 10-item Center for Epidemiological Studies Depression Scale (CESD-10) in CHARLS is a simplified version of the depression scale. Each participant’s depression scores in each wave were evaluated using CESD-10. Depression was treated as a dichotomous variable, in that a score greater than or equal to 10 was considered to indicate depression, and a score below 10 was considered normal [23].

#### Socioeconomic groups

Educational attainment and annual per-capita household consumption expenditure were used as proxies for socioeconomic status. Different socioeconomic groups were defined based on educational attainment (time-invariant, including illiterate; primary school; secondary school and above) and quartiles of per-capita household consumption expenditure (quartile 1 for the most deprived and quartile 4 for the most affluent).

#### Covariates

The following variables were included as covariates: age, sex, marital status (married/partnered, and single/others), educational attainment (illiterate, primary school, secondary school and above), residence (rural, and urban), annual per-capita household consumption expenditure (quartiles 1–4), health insurance (public insurance, and others), geographic locations (east, middle, and west), activities of daily living (ADLs, impaired, and unimpaired), instrumental activities of daily living (IADLs, impaired, and unimpaired). Sex and geographic locations were time-invariant variables.

### Statistical analysis

First, we conducted a descriptive statistical analysis of study participants from 2011 to 2018. Continuous variables were represented by means and standard deviation (SD), and classified variables were measured by frequencies and percentages. Prevalence was used to measure the trends of diseases. The prevalence of the exact 13 self-reported non-communicable diseases was shown in Supplementary Table 1.

Next, a panel data approach of random-effects linear regression was used to examine the associations between socioeconomic status and the number of non-communicable diseases. Random-effects negative binomial regression models were employed to investigate the association between the number of non-communicable diseases and body pain. For the negative binomial regression analysis, incidence rate ratios (IRRs) and 95% confidence intervals (CIs) were reported. Random-effects linear regression models were used to estimate the association between the number of non-communicable diseases and sleep duration.

Random-effects logistic regression models were used to estimate the association between the number of non-communicable diseases and the likelihood of developing depression. Specifically, the mediating effects of the number of body pain and sleep duration on the correlations between the number of non-communicable diseases and depression were examined by a stepwise regression method [24]. Odds ratios (ORs) and 95% CIs were reported. To explore the differential associations between the number of non-communicable diseases and depression in population groups, subgroup analyses stratified by socioeconomic status were performed, using the same regression analyses but removing stratified variables. A *p* values less than 0.05 was considered as significant.

To address the potential biases introduced by the overlapping individuals, we employed random-effects models, which accounted for the longitudinal nature of the data and the repeated observations of the same individuals across different waves. To consider the differences in follow-up time and loss to follow-up, we used multiple imputation techniques to estimate the missing values and create a more complete dataset for analysis. By estimating the missing values based on other observed variables, we can ensure that the results are comparable across different waves of the study. In addition, to account for the survey design, stratification, and clustering, we incorporated sampling weights in the analysis methods to ensure accurate representation of the population prevalence and unbiased estimation of parameters. All statistical analyses were performed using Stata (version 16.0).

## Results

### Descriptive statistics

Socio-demographic characteristics of study participants in 2011–2018 are shown in Table 1. The mean age of participants was 58.44 years (SD = 0.08) at 2011 baseline. The proportion of people with multimorbidity seemed to increase substantially with age. In 2011, the number of people with multimorbidity was 6,412, accounting for 37.87% of the total population. In 2018, the proportion of people with multimorbidity increased to 61.14%. The average number of body pain per individual was 1.27 (SD = 0.02) in 2011 and rose to 2.92 (SD = 0.03) in 2018. People slept for an average of 6.88 h (SD = 0.02) per day and 5,335 (34.69%) had depression in 2011.

**Table 1:** Socio-demographic characteristics of study participants aged 45 years and older in China, 2011–2018

Characteristics	Wave 2011 (*N* = 16,931)	Wave 2013 (*N* = 14,284)	Wave 2015 (*N* = 13,902)	Wave 2018 (*N* = 12,344)
Multimorbidity	6,412 (37.87%)	6,381 (43.68%)	7,853 (56.02%)	7,981 (61.14%)
Number of body pain	1.27 (0.02)	0.93 (0.02)	1.66 (0.03)	2.92 (0.03)
Sleep duration	6.88 (0.02)	6.81 (0.02)	6.99 (0.02)	6.92 (0.02)
Depression				
No	10,047 (65.31%)	9,002 (69.72%)	8,767 (67.16%)	7,140 (63.11%)
Yes	5,335 (34.69%)	3,909 (30.28%)	4,286 (32.84%)	4,174 (36.89%)
Age (years)	58.44 (0.08)	61.46 (0.08)	63.02 (0.08)	65.65 (0.08)
Sex				
Male	8,201 (48.44%)	6,935 (48.55%)	6,716 (48.31%)	5,953 (48.22%)
Female	8,730 (51.56%)	7,349 (51.45%)	7,186 (51.69%)	6,391 (51.78%)
Marital status				
Single	2,493 (14.73%)	2,292 (16.04%)	2,333 (16.78%)	2,351 (19.04%)
Married/partnered	14,428 (85.27%)	11,990 (83.96%)	11,569 (83.22%)	9,993 (80.96%)
Educational attainment				
Illiterate	7,268 (42.94%)	6,338 (44.37%)	6,085 (43.77%)	5,310 (43.02%)
Primary school	3,466 (20.48%)	2,970 (20.80%)	2,884 (20.75%)	2,607 (21.12%)
Secondary school and above	6,191 (36.58%)	4,976 (34.83%)	4,933 (35.48%)	4,427 (35.86%)
Residence status				
Rural	8,469 (50.02%)	7,694 (53.87%)	7,649 (55.02%)	6,843 (55.44%)
Urban	8,462 (49.98%)	6,590 (46.13%)	6,253 (44.98%)	5,501 (44.56%)
Health insurance				
Private insurance/others	1,418 (8.49%)	706 (5.00%)	1,368 (9.88%)	501 (4.07%)
Public insurance	15,290 (91.51%)	13,393 (95.00%)	12,470 (90.12%)	11,831 (95.93%)
Geographic locations				
East locations	7,510 (44.36%)	6,242 (43.70%)	5,911 (42.52%)	5,193 (42.07%)
Middle locations	4,355 (25.72%)	3,807 (26.65%)	3,783 (27.22%)	3,405 (27.59%)
West locations	5,066 (29.92%)	4,235 (29.65%)	4,207 (30.26%)	3,746 (30.34%)
Household consumption quartiles				
Quartile 1 (the most deprived)	3,149 (22.32%)	2,267 (24.18%)	2,453 (26.55%)	2,752 (26.91%)
Quartile 2	3,159 (22.39%)	2,318 (24.73%)	2,400 (25.98%)	2,572 (25.15%)
Quartile 3	3,431 (24.32%)	2,400 (25.60%)	2,180 (23.60%)	2,548 (24.91%)
Quartile 4 (the most affluent)	4,368 (30.96%)	2,390 (25.49%)	2,206 (23.88%)	2,356 (23.03%)
ADLs				
Unimpaired	13,960 (84.04%)	11,567 (81.92%)	10,762 (77.96%)	9,601 (77.98%)
Impaired	2,651 (15.96%)	2,552 (18.08%)	3,043 (22.04%)	2,710 (22.02%)
IADLs				
Unimpaired	13,318 (79.34%)	10,788 (76.14%)	10,223 (73.83%)	8,901 (72.29%)
Impaired	3,469 (20.66%)	3,381 (23.86%)	3,624 (26.17%)	3,411 (27.71%)

ADLs = activities of daily living, IADLs = instrumental activities of daily living

**Fig. 1:**
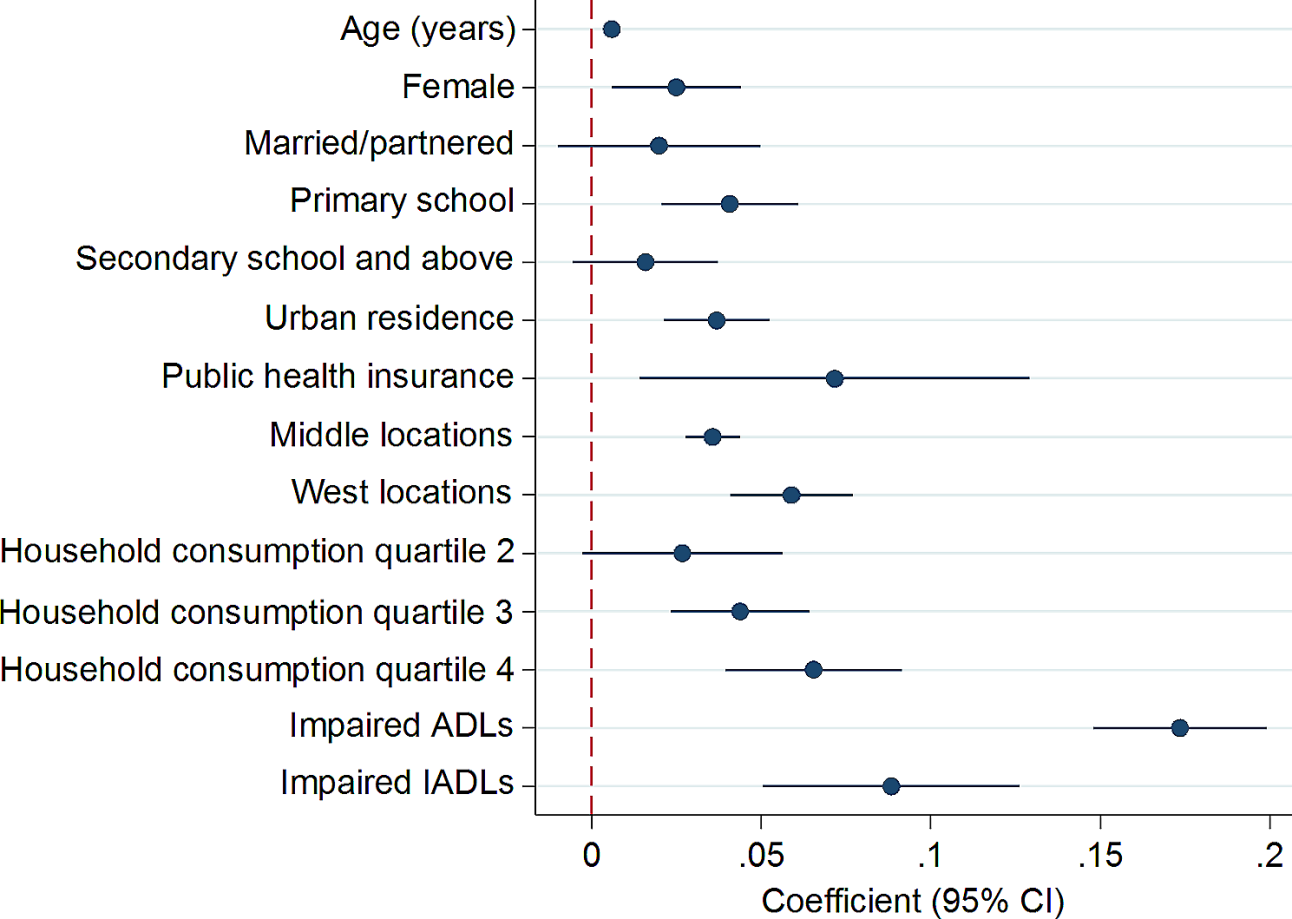
Longitudinal analysis of determinants of multimorbidity among people aged 45 years and older in China, 2011–2018. ADLs = activities of daily living, IADLs = instrumental activities of daily living. This table presents the results of the longitudinal analysis using data from four waves of CHARLS (2011–2018) to examine the associations between sociodemographic variables, ADLs, IADLs, and multimorbidity over time. The longitudinal aspect of the analysis accounts for the trends and patterns in multimorbidity determinants among the study population

In Fig. 1 and Supplementary Table 2, the probability of having multimorbidity increased with age (β = 0.006, 95% CI = 0.004–0.007). It also increased with higher socioeconomic status. To be exact, participants with primary school education (β = 0.041, 95% CI = 0.020–0.061) and higher household consumption quartiles (β = 0.044, 95% CI = 0.023–0.064 for household consumption quartile 3 and β = 0.065, 95% CI = 0.039–0.091 for household consumption quartile 4) were more likely to have multimorbidity.

### Multimorbidity, body pain, sleep duration and depression

Table 2 shows the associations of multimorbidity with the number of body pain and sleep duration among people aged 45 years and older in China from 2011 to 2018. Multimorbidity was associated with increased number of body pain (incidence rate ratio (IRR) = 1.53, 95% CI = 1.45–1.61) and decreased sleep duration (β=-0.26, 95% CI=-0.36–-0.15). Those with higher educational attainment had more body pain (IRR = 1.20, 95% CI = 1.09–1.33 for those with primary education; IRR = 1.21, 95% CI = 1.09–1.34 for those with secondary school and above). Higher household consumption quartiles were correlated with more body pain (IRR = 1.05, 95% CI = 1.00–1.10 for those in household consumption quartile 2; IRR = 1.11, 95% CI = 1.05–1.17 for those in household consumption quartile 3; IRR = 1.09, 95% CI = 1.03–1.15 for those in household consumption quartile 4).

**Table 2:** The associations of multimorbidity with the number of body pain and sleep duration among people aged 45 years and older in China, 2011–2018

	The number of body pain		Sleep duration	
	IRR (95% CI)	*P* value	Coefficient (95% CI)	*P* value
Multimorbidity	1.53 (1.45, 1.61)	< 0.001	-0.26 (-0.36, -0.15)	0.004
Age (years)	1.05 (1.04, 1.05)	< 0.001	0.01 (-0.01, 0.03)	0.357
Female	1.39 (1.28, 1.51)	< 0.001	-0.39 (-0.50, -0.28)	0.001
Married/partnered	0.98 (0.90, 1.06)	0.587	0.12 (0.05, 0.18)	0.011
Primary school	1.20 (1.09, 1.33)	< 0.001	0.10 (-0.02, 0.22)	0.078
Secondary school and above	1.21 (1.09, 1.34)	< 0.001	0.09 (-0.06, 0.25)	0.148
Urban residence	0.78 (0.72, 0.85)	< 0.001	-0.05 (-0.18, 0.08)	0.303
Public health insurance	1.11 (1.03, 1.20)	0.009	0.04 (-0.15, 0.22)	0.586
Middle locations	1.11 (1.01, 1.23)	0.034	-0.06 (-0.21, 0.08)	0.271
West locations	1.06 (0.97, 1.16)	0.198	-0.26 (-0.31, -0.22)	< 0.001
Household consumption quartile 2	1.05 (1.00, 1.10)	0.049	-0.03 (-0.20, 0.14)	0.608
Household consumption quartile 3	1.11 (1.05, 1.17)	< 0.001	-0.04 (-0.09, 0.02)	0.139
Household consumption quartile 4 (the most affluent)	1.09 (1.03, 1.15)	0.004	0.005 (-0.10, 0.11)	0.890
Impaired ADLs	1.53 (1.46, 1.60)	< 0.001	-0.41 (-0.60, -0.21)	0.008
Impaired IADLs	1.36 (1.30, 1.42)	< 0.001	-0.16 (-0.36, 0.03)	0.073

IRR = incidence rate ratio, CI = confidence interval, ADLs = activities of daily living, IADLs = instrumental activities of daily living

Table 3 shows the associations between multimorbidity and depression, mediated by the number of body pain and sleep duration. In Model 1, multimorbidity was associated with higher risks of depression (OR = 1.97, 95% CI = 1.85–2.10). When adjusting for the number of body pain and sleep duration stepwise (Model 2 and Model 3), more body pain (OR = 1.19, 95% CI = 1.18–1.20 in Model 2) and shorter sleep duration (OR = 0.83, 95% CI = 0.82–0.84 in Model 3) was associated with higher risks of depression. The associations between multimorbidity and depression persisted but the value of OR decreased to 1.58 (95% CI = 1.48–1.68) in Model 2 and 1.54 (95% CI = 1.44–1.64) in Model 3, which to some extent validated the mediating effects of the number of body pain and sleep duration.

**Table 3:** The associations between multimorbidity and depression: the mediating effect of the number of body pain and sleep duration, among people aged 45 years and older in China, 2011–2018

	Model 1		Model 2		Model 3	
	OR (95% CI)	*P* value	OR (95% CI)	*P* value	OR (95% CI)	*P* value
Multimorbidity	1.97 (1.85, 2.10)	< 0.001	1.58 (1.48, 1.68)	< 0.001	1.54 (1.44, 1.64)	< 0.001
Age (years)	0.98 (0.97, 0.98)	< 0.001	0.98 (0.97, 0.98)	< 0.001	0.98 (0.97, 0.98)	< 0.001
Female	1.71 (1.59, 1.84)	< 0.001	1.49 (1.39, 1.61)	< 0.001	1.40 (1.31, 1.51)	< 0.001
Married/partnered	0.61 (0.55, 0.67)	< 0.001	0.61 (0.55, 0.67)	< 0.001	0.63 (0.58, 0.70)	< 0.001
Primary school	0.85 (0.77, 0.93)	< 0.001	0.86 (0.79, 0.94)	0.001	0.88 (0.80, 0.96)	0.004
Secondary school and above	0.58 (0.53, 0.64)	< 0.001	0.60 (0.55, 0.66)	< 0.001	0.62 (0.57, 0.68)	< 0.001
Urban residence	0.66 (0.61, 0.71)	< 0.001	0.71 (0.66, 0.77)	< 0.001	0.70 (0.65, 0.76)	< 0.001
Public health insurance	0.91 (0.80, 1.02)	0.111	0.89 (0.79, 1.00)	0.049	0.87 (0.77, 0.98)	0.019
Middle locations	1.57 (1.44, 1.71)	< 0.001	1.49 (1.37, 1.62)	< 0.001	1.47 (1.36, 1.60)	< 0.001
West locations	1.95 (1.79, 2.12)	< 0.001	1.73 (1.60, 1.88)	< 0.001	1.66 (1.53, 1.80)	< 0.001
Socioeconomic Status Class 2	0.97 (0.90, 1.04)	0.397	0.95 (0.88, 1.03)	0.209	0.95 (0.88, 1.03)	0.183
Socioeconomic Status Class 3	0.90 (0.83, 0.98)	0.012	0.88 (0.81, 0.95)	0.002	0.87 (0.80, 0.94)	0.001
Socioeconomic Status Class 4 (the most affluent)	0.84 (0.77, 0.92)	< 0.001	0.83 (0.76, 0.90)	< 0.001	0.82 (0.75, 0.89)	< 0.001
Impaired ADLs	2.71 (2.50, 2.94)	< 0.001	2.16 (1.99, 2.34)	< 0.001	2.07 (1.91, 2.25)	< 0.001
Impaired IADLs	2.21 (2.05, 2.38)	< 0.001	1.97 (1.83, 2.12)	< 0.001	1.97 (1.83, 2.13)	< 0.001
The number of body pain			1.19 (1.18, 1.20)	< 0.001	1.18 (1.16, 1.19)	< 0.001
Sleep duration					0.83 (0.82, 0.84)	< 0.001

OR = odds ratio, CI = confidence interval, ADLs = activities of daily living, IADLs = instrumental activities of daily living

### Subgroup analysis by socioeconomic status

**Fig. 2:**
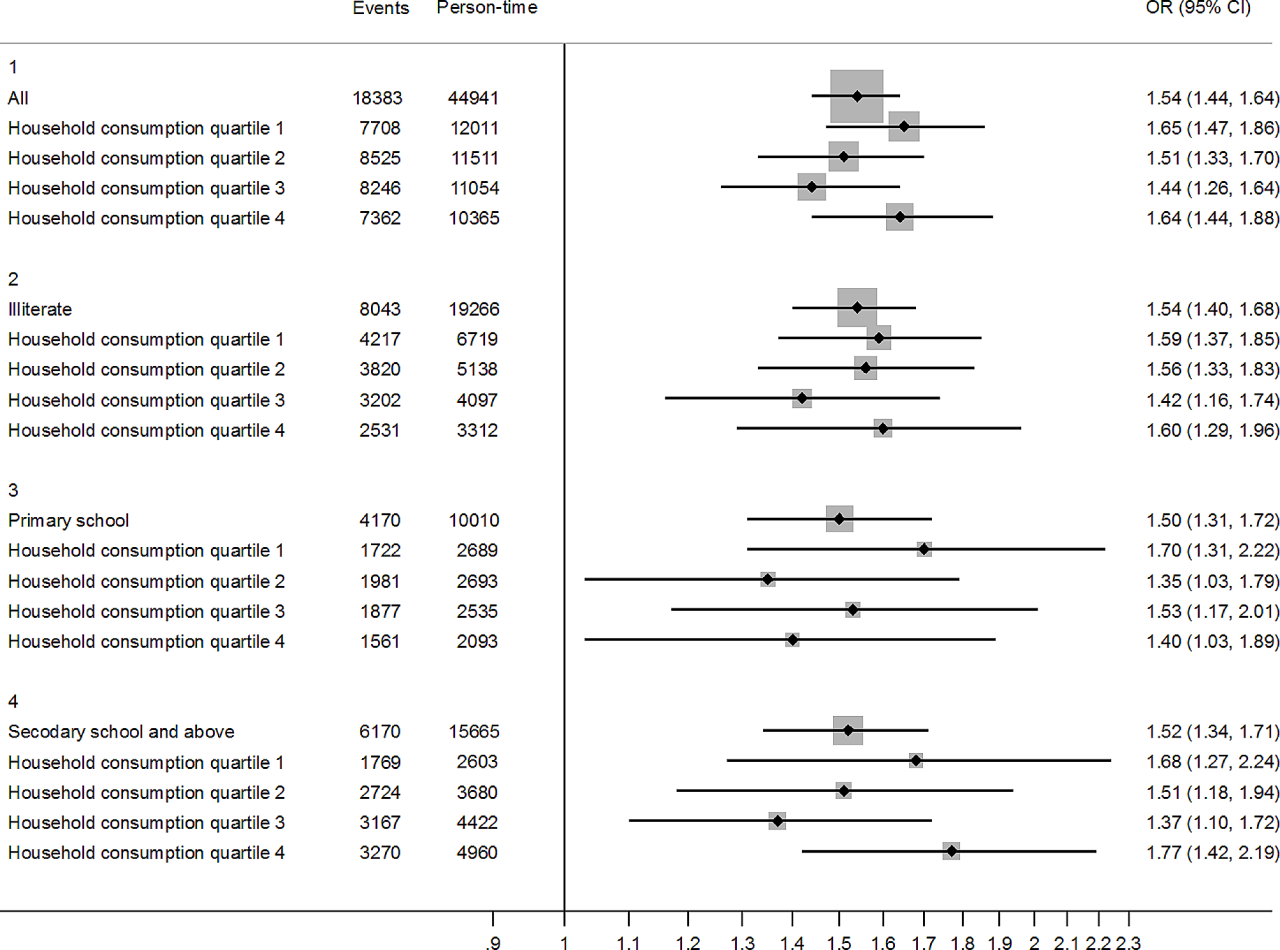
The associations between multimorbidity and depression by socioeconomic status class among people aged 45 years and older in China, 2011–2018. OR = odds ratio, CI = confidence interval

In Fig. 2, the associations between multimorbidity and depression were similar across socioeconomic groups—i.e., for each group classified by educational attainment, those with multimorbidity had higher risk of depression (odds ratio (OR) = 1.54, 95% CI = 1.40–1.68 for those illiterate; OR = 1.50, 95% CI = 1.31–1.72 for those with primary education; OR = 1.52, 95% CI = 1.34–1.71 for those with secondary education and above). Moreover, the associations between multimorbidity and depression were similar across household consumption quartiles. The associations persisted even in the highest household consumption quartile and those with an educational attainment of secondary school and above (OR = 1.77, 95% CI = 1.42–2.19).

## Discussion

To the best of our knowledge, this study is the first panel data analysis from a nationally representative longitudinal survey of middle-aged and older Chinese people to examine the associations of multimorbidity with body pain, sleep duration, and depression; and whether the associations varied by socioeconomic status. It was found that multimorbidity was common among Chinese people aged 45 years and older. An additional chronic non-communicable disease was associated with increased body pain, and decreased sleep duration. Furthermore, multimorbidity was associated with a significantly increased likelihood of depression, with the mediating effects of the number of body pain and sleep duration. The associations between multimorbidity and depression persisted among different socioeconomic groups classified by educational attainment and quartiles of household consumption expenditure.

For any individual, the likelihood of multimorbidity increased with age, given that chronic conditions might not be resolved. It was found in our study that the number of multimorbidity also increased with higher quartiles of household consumption and educational attainment. It contrasted with evidence from high-income countries that showed an increased prevalence of multimorbidity in populations with low socioeconomic status [25, 26]. In China, people in higher-income groups could have better access to healthcare services and better health literacy, and they are more likely to be diagnosed (or even over-diagnosed) with non-communicable diseases than those in lower-income groups [27, 28]. Furthermore, recall of specific chronic conditions diagnosed in the past might be improved for them, for example, because they are paying for ongoing treatment. In addition, low capacity and accessibility of rural facilities may mean that physical multimorbidity is under-reported in rural areas.

Further, it was found that more chronic non-communicable diseases were associated with increased body pain and decreased sleep duration. Body pain is one of the major symptoms of chronic diseases. Chronic diseases, such as arthritis, rheumatism, and gastrointestinal diseases, are often accompanied by pain symptoms [29]. Chronic diseases are long-course diseases, so the pain caused is also long term. Patients with multiple chronic conditions may experience even greater levels of pain. Chronic diseases and their associated pain and discomforts can also interfere with sleep [30]. Conversely, sleep disorders can exacerbate chronic conditions and contribute to the experience of pain [31]. A recent meta-analysis estimated that 44% of patients with chronic pain had sleep disturbances; insomnia (72%), restless leg syndrome (32%) and obstructive sleep apnea (32%) were the most common diagnoses [32].

Multimorbidity was further associated with a significantly increased likelihood of depression, mediated by the number of body pain and sleep duration. This means that individuals with multimorbidity were more likely to experience depression, and this increased risk could be partially explained by the presence of body pain and sleep disturbances. People with chronic diseases may experience physical dysfunction that adversely impacts daily life activities. Older people are prone to feel pain, anxiety, and even despair when they encounter difficulties in daily life [33]. The pain caused by physical discomfort can also result in irritability, restlessness, and other negative emotions, which further lead to the occurrence of depression [17, 34]. Sleep restriction would also promote the development of depressive symptoms [35]. Good sleep helps with self-regulation, which can help ease depressive symptoms [36].

The associations between multimorbidity and depression appeared to be similar across different socioeconomic groups. Our findings were in part consistent with other studies examining physical multimorbidity in LMICs, such as India and Bangladesh, which found that obesity, physical inactivity, and consumption of tobacco, alcohol, fat, salt, and processed food were prevalent among high socioeconomic groups [27, 37]. Thus, the associations of multimorbidity with body pain, sleep duration, and depression could persist among people of higher socioeconomic status. On the other hand, people of lower socioeconomic status generally have limited access to healthcare, financial constraints, and increased stress levels. Additionally, lifestyle factors and a lack of awareness or education about health management may exacerbate these associations, perpetuating health disparities among this vulnerable population.

### Strengths and limitations

This study presented valuable findings that can inform public health policies and interventions. First, our findings provided new evidence to inform the development of targeted policies and interventions to tackle the increasing burden of multimorbidity and its negative impacts in China. It underscored the importance of a comprehensive approach to healthcare. Public health professionals, policymakers, and healthcare providers should work together to ensure that programs and services address not only the individual health conditions but also their coexistence and interconnected effects on overall well-being. In addition, it highlighted the importance of addressing health disparities. Public health initiatives should focus on creating equal access to healthcare services, especially for individuals with lower socioeconomic status, to ensure they effectively manage their chronic conditions and mitigate risks associated with multimorbidity.

Our study had several limitations. First, the use of self-reported measures of chronic disease may underestimated their prevalence, particularly among older people and those from lower socioeconomic and educational backgrounds. Second, the CHARLS questionnaire did not include all typical chronic diseases in clinical database studies. Further research is also warranted on the effect of multimorbidity caused by infectious diseases (e.g., tuberculosis, AIDS, COVID-19). Third, we aggregated all diseases into a single count variable and did not include individual diseases in the analysis, which might overlook potential variations in the effects of specific diseases on the outcomes of interest, and the unique contributions of individual diseases might be masked. Fourth, psychological distress as a subjective experience of discomfort, is often confused with psychiatric disorders [38]. Therefore, apart from exploring depression, a common clinical psychological disorder, future studies are needed to also assess the impact of multimorbidity on the subjective perception of distress. Finally, this study only included people from China aged 45 years and older in 2011. The multimorbidity and its effect among younger populations can be considered in future studies.

## Conclusions

In conclusion, multimorbidity was associated with increased body pain, decreased sleep duration, and further led to increased depression risks. China, with the largest ageing population in the world, should pay attention to the multimorbidity of middle-aged and older adults. It is also important for policymakers and healthcare providers to develop effective and sustainable interventions to relieve their body pain, ensure sufficient sleep, so as to further reduce depression risks.

### Electronic supplementary material

Below is the link to the electronic supplementary material.

Supplementary Material 1

## Data Availability

The data supporting the conclusion of this article are includes within the article. Any queries regarding these data may be directed to the corresponding author.
