# Supplementary material for: Associations of multimorbidity with body pain, sleep duration, and depression among middle-aged and older adults in China
Source: Health Qual Life Outcomes. 2024 Feb 27;22:23. doi: 10.1186/s12955-024-02238-x (PMC10900654; doi:10.1186/s12955-024-02238-x)
Supplement: Supplementary file 1 — Supplementary Material 1 [file 12955_2024_2238_MOESM1_ESM.docx]

**Supplementary Materials**

**Supplementary Table 1** Prevalence of the exact 13 self-reported non-communicable diseases for study participants aged 45 years and older in China, 2011–2018

| **Non-communicable diseases** | **Wave 2011 (N=16,931)** | **Wave 2013 (N=14,284)** | **Wave 2015 (N=13,902)** | **Wave 2018 (N=12,344)** |
| --- | --- | --- | --- | --- |
| Diabetes | 1,093 (6.56%) | 1,156 (8.32%) | 1,443 (10.71%) | 1,754 (14.22%) |
| Hypertension | 4,569 (27.34%) | 4,252 (30.34%) | 4,919 (36.31%) | 5,338 (43.28%) |
| Dyslipidemia | 1,769 (10.74%) | 1,944 (14.30%) | 2,701 (20.40%) | 2,995 (24.29%) |
| Heart disease | 2,059 (12.32%) | 1,987 (14.19%) | 2,605 (19.20%) | 2,708 (21.96%) |
| Stroke | 501 (2.99%) | 507 (3.61%) | 632 (4.63%) | 1,119 (9.07%) |
| Cancer | 151 (0.90%) | 162 (1.16%) | 237 (1.74%) | 318 (2.57%) |
| Chronic lung disease | 1,597 (9.54%) | 1,557 (11.11%) | 2,089 (15.31%) | 2,175 (17.63%) |
| Digestive disease | 3,490 (20.83%) | 3,317 (23.58%) | 4,269 (31.32%) | 3,907 (31.68%) |
| Liver disease | 612 (3.67%) | 712 (5.10%) | 986 (7.27%) | 954 (7.73%) |
| Kidney disease | 941 (5.64%) | 962 (6.89%) | 1,455 (10.72%) | 1,358 (11.00%) |
| Arthritis | 5,330 (31.80%) | 4963 (35.21%) | 6,109 (44.84%) | 5,260 (42.66%) |
| Asthma | 723 (4.32%) | 717 (5.10%) | 892 (6.53%) | 873 (7.07%) |
| Psychological problems | 270 (1.62%) | 250 (1.78%) | 371 (2.72%) | 462 (3.74%) |

**Supplementary Table 2** Longitudinal analysis of determinants of multimorbidity among people aged 45 years and older in China, 2011–2018

|  | Coefficient (95% CI) | ***P*** value |
| --- | --- | --- |
| Age (years) | 0.006 (0.004, 0.007) | .001 |
| Female | 0.025 (0.006, 0.044) | .025 |
| Married/partnered | 0.020 (-0.010, 0.050) | .126 |
| Primary school | 0.041 (0.020, 0.061) | .008 |
| Secondary school and above | 0.016 (-0.006, 0.037) | .101 |
| Urban residence | 0.037 (0.021, 0.052) | .005 |
| Public health insurance | 0.072 (0.014, 0.129) | .029 |
| Middle locations | 0.036 (0.027, 0.044) | .001 |
| West locations | 0.059 (0.041, 0.077) | .002 |
| Household consumption quartile 2 | 0.027 (-0.003, 0.056) | .064 |
| Household consumption quartile 3 | 0.044 (0.023, 0.064) | .007 |
| Household consumption quartile 4 (the most affluent) | 0.065 (0.039, 0.091) | .004 |
| Impaired ADLs | 0.173 (0.148, 1.199) | <.001 |
| Impaired IADLs | 0.088 (0.050, 0.126) | .005 |

This table presents the results of the longitudinal analysis using data from four waves of CHARLS (2011–2018) to examine the associations between sociodemographic variables, ADLs, IADLs, and multimorbidity over time. The longitudinal aspect of the analysis accounts for the trends and patterns in multimorbidity determinants among the study population.
